# Graph theory reveals amygdala modules consistent with its anatomical subdivisions

**DOI:** 10.1038/s41598-017-14613-4

**Published:** 2017-10-31

**Authors:** Elisabeth C. Caparelli, Thomas J. Ross, Hong Gu, Xia Liang, Elliot A. Stein, Yihong Yang

**Affiliations:** 1 Neuroimaging Research Branch, National Institute on Drug Abuse, National Institutes of Health, Baltimore, Maryland USA; 2 Research Center of Basic Space Science, Harbin Institute of Technology, Harbin, China

## Abstract

Similarities on the cellular and neurochemical composition of the amygdaloid subnuclei suggests their clustering into subunits that exhibit unique functional organization. The topological principle of community structure has been used to identify functional subnetworks in neuroimaging data that reflect the brain effective organization. Here we used modularity to investigate the organization of the amygdala using resting state functional magnetic resonance imaging (rsfMRI) data. Our goal was to determine whether such topological organization would reliably reflect the known neurobiology of individual amygdaloid nuclei, allowing for human imaging studies to accurately reflect the underlying neurobiology. Modularity analysis identified amygdaloid elements consistent with the main anatomical subdivisions of the amygdala that embody distinct functional and structural properties. Additionally, functional connectivity pathways of these subunits and their correlation with task-induced amygdala activation revealed distinct functional profiles consistent with the neurobiology of the amygdala nuclei. These modularity findings corroborate the structure–function relationship between amygdala anatomical substructures, supporting the use of network analysis techniques to generate biologically meaningful partitions of brain structures.

## Introduction

The amygdala is involved in a series of emotional processes and cognitive functions including learning, memory, attention and perception^1^. Although small in total size, its complex composition of structurally and functionally heterogeneous subnuclei supports these multiple functions. Histological studies suggest that the amygdala may be composed of at least twenty subnuclei^2^ that share cytoarchitecture, myeloarchitecture and chemoarchitecture features. Based on these similarities, schemas have been proposed to segregate these nuclei into subunits based on their functional and anatomical characteristic^3^. Although the boundaries and even the names of these subunits remain unsettled, one of the most widely accepted classification schemas classifies them as the superficial (SF) (corticoid) amygdaloid nucleus, the centromedial (CM) group and the laterobasal (LB) complex^4–6^. A stereotaxic probabilistic map of the human amygdala, based on the superposition of cytoarchitectonic mapping of cell-body stained histological sections, has been developed by Amunts and colleagues^5^ and classifies the SF group to include the anterior amygdaloid area, the amygdalopyriform transition area, the amygdaloid-hippocampal area and cortical nuclei (intermediate, dorsal, ventral and posterior), the CM group combines the central and medial nuclei, while the LB group incorporates the lateral, basolateral, basomedial and paralaminar nuclei. This mapping is quite consistent with the cellular and neurochemical composition of the amygdaloid nuclei, since the CM, which is continuous with the bed nucleus of stria terminals, is comprised of cells that are morphologically similar to those in the striatum, while the LB and SF resemble cortical area^7–10^. Additionally, the LB is the main amygdala division that receives efferent projections from basal forebrain cholinergic neurons projecting mostly to the basolateral nucleus^4,11^.

The neurobiological composition of these main subdivisions indicates a potentially exclusive functional/anatomical neural interaction that may be captured either in synchronized fluctuation of the fMRI signal, as in rsfMRI, or through anatomical connections, such as, diffusion tensor imaging (DTI). For this reason, different functional and structural MRI based approaches have been proposed to parse the amygdala architecture. As a result, while some studies found the amygdala subdivided in two main regions, which were identified as superior^12^/superficial-cluster^13^ and inferior^12^/deep-cluster^13^, representing the LB and smaller nuclei (inferior/deep-cluster) and the CM and cortical nuclei (superior/superficial-cluster)^14^, others found the amygdala subdivided into four main subregions, dividing LB into lateral and basal regions and the CM into central and medial, leaving the cortical nuclei undifferentiated^15^. Therefore, these findings are not fully in agreement with the amygdala subdivisions shown in the stereotaxic probabilistic map^5^.

Two recent studies^16,17^, using imaging approaches, found three subdivisions of the amygdala forecasted by the probabilistic atlas^5^. The first study proposed a method that started from a hypothetical topographic model, based on the functional-anatomic organization of brain networks subserving social cognition, by defining three social networks: perception, which would be associated with the ventrolateral sector of the amygdala; affiliation, related to the medial section of the amygdala; and aversion, linked with the dorsal sector of the amygdala^16^. As a result, three amygdala regions were pre-defined in those locations (spherical ROIs) and using an iterative seed-target-seed methodology, the border of these subdivisions were established. The second study proposed an imaging-parcellation method based on meta-analysis, where three amygdala subdivisions were identified by computing whole-brain co-activation patterns for each amygdala seed voxel and grouping these seeds based on similarities between their co-activation patterns^17^. This method required a systematic analysis of a broad range of associated experiments (6,500 fMRI and PET studies) to increase the robustness of parcellation results and to avoid dependence on any particular user-specified parameter. Therefore, while the first approach^16^ predefine the initial number of subdivisions, the second approach was a meta-analysis requiring a significant amount of data from different imaging sources.

In this work, we propose to use resting state BOLD data and the topological principle of community structure to identify the main subdivisions of the amygdala. Our approach is based on global topological characteristics that has been shown to produce stable brain structural and functional networks, suggesting that the observed hierarchical modular organization coincide with the underlying anatomical communities at different scales^18^. Furthermore, by using hierarchical modularity analysis to explore the community structure of brain networks^19,20^, previous studies have shown that functionally and anatomically related brain regions are more densely interconnected, with relatively few connections between these clusters, indicating that these networks are intimately related and share common topological features, such as modules and hubs^21^. Therefore, based on these concepts, we employed the principle of modularity to identify subnetworks within the amygdala. We hypothesized that the modular organization of the amygdala will reflect its internal functional segregation and integration consistent with the neurobiological properties of its nuclei. For this purpose, we applied the community structure algorithm to a high spatial and temporal resolution rsfMRI data from the Human Connectome Project (HCP)^22,23^. We further assessed the functional consequence of each identified subnetwork by determining their functional connectivity and then by correlating these circuits with amygdala activation during an emotional processing task. Our findings validate the association between the modular structure of the amygdala and the biological characteristics of amygdala subdivisions.

## Results

### Modularity

**Figure 1:**
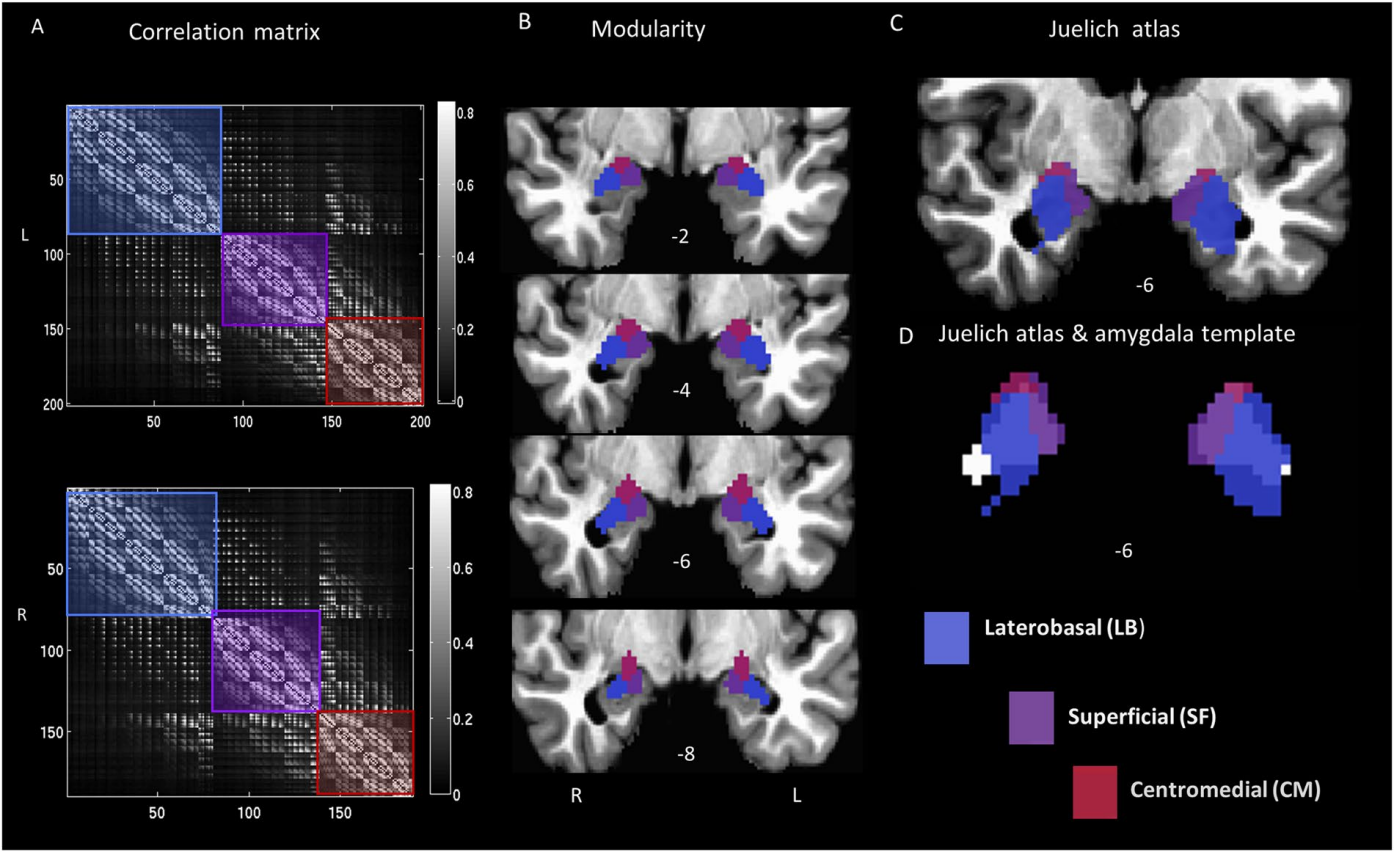
Modularity results for the amygdala subdivisions. (**A**) Correlation matrix for the amygdala template with modules overlaid in semi-transparent colors, greyscale values indicate r-values, (**B**) modularity results: LBL (696 mm^3^), LBR (640 mm^3^), SFL (472 mm^3^), SFR (464 mm^3^), CML (440 mm^3^), CMR (408 mm^3^), are displayed on coronal slices located at y-axis values: -2, -4, -6, -8 (L/R suffix = left/right). The maximum modularity factors obtained for this parcellation were Q_L_ = 0.26, Q_R_ = 0.25; (**C**) Juelich atlas overlaid on a coronal anatomical image (**D**) Juelich atlas superimposed on the amygdala template (shown in white, under the Juelich atlas); radiological convention.

Modular analysis of the amygdala revealed three distinct modules (Fig. 1A and B) that are consistent with the probabilistic anatomical subregion maps^5^, displayed in the Juelich histological atlas (50% probabilistic mask, FSL - Fig. 1C). However, the borders of the atlas, as implemented in FSL, do not completely coincide with macro-anatomical landmarks of the amygdala^5^ (Fig. 1D; Juelich atlas volume, left: 2968 mm^3^, right: 2904 mm^3^ ^6^, making direct comparison of the modularity results and the anatomical subdivisions difficult, since the amygdala template is smaller (left: 1608 mm^3^, right: 1512 mm^3^), but better represents the average amygdala size in healthy adults^24^. Despite size discrepancies, the synergy between the relative size of the subdivisions found in the Juelich atlas (LB > SF > CM) and the modules obtained from our modularity approach (lateral > medial > dorsal), together with the closeness between the subdivision location, suggest labeling the dorsal module as CM, the medial as SF and the lateral as LB.

### FC maps

**Figure 2:**
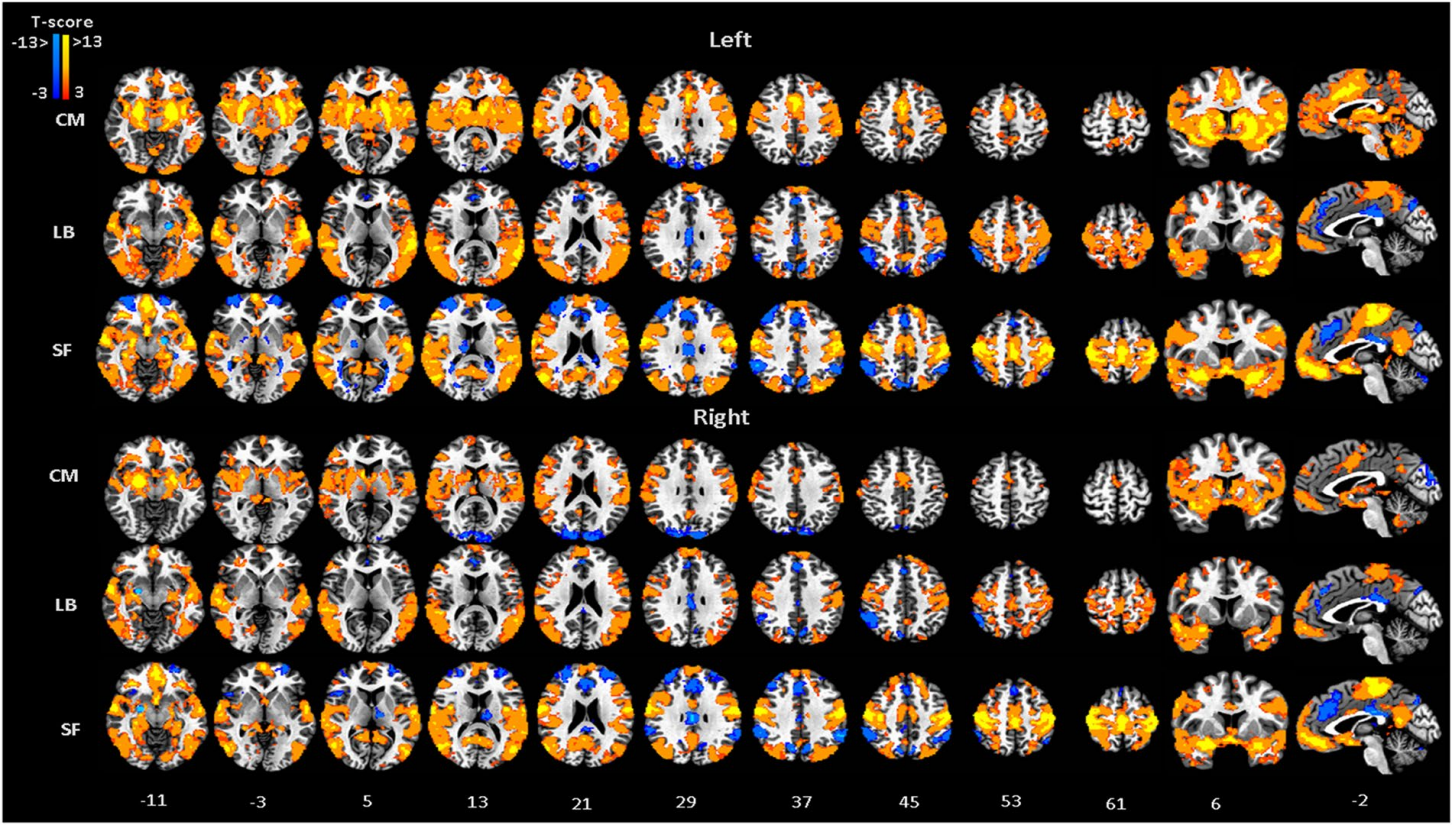
FC maps for the amygdala subdivisions, CM, LB and SF, obtained from modularity results. MNI standard space; radiological convention; significance: p < 0.05 FWE corrected. Left/Right indicate seed locations.

**Figure 3:**
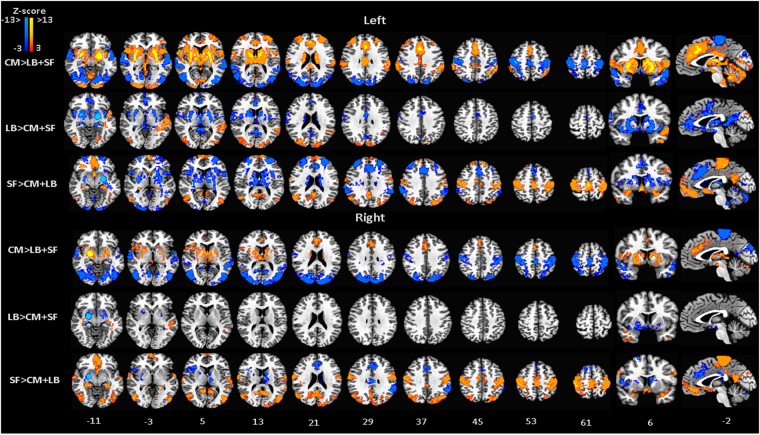
FC-differential maps contrasting the connectivity map of each amygdala subdivision, CM, LB and SF, against the average connectivity pattern of the other two subdivisions, LB + SF, CM + SF, CM + LB. MNI standard space; radiological convention; significance: p < 0.05 FWE corrected.

Patterns of significant correlation for each amygdala subdivision reveal unique connectivity circuits. MRI signal at the CM was mainly correlated with the signal in the middle and anterior cingulate cortices, frontal cortex, striatum, insula, cerebellum and precuneus, while a negative correlation was observed at the occipital gyrus (Fig. 2, Table 1). However, an overall reduction in the connectivity strength was observed for the right when compared with the left CM, which was not seen for other amygdala subdivisions (Figs 2 and 2S). In spite of this reduction in strength, the connectivity pattern is preserved between the two sides of this amygdala subdivision. Direct comparison with correlation results from the other seed regions highlight this unique path of connections when contrasting the functional connectivity results of the CM with either the combined results from LB and SF (Fig. 3, Table 2) or the connectivity results from each of the others subdivisions (Fig. 1S, Table 1S).

**Table 1:** Cluster locations for the functional connectivity maps calculated for each amygdala subdivision. T-test results from partial correlation for each subset are corrected for multiple comparisons, *p*_corrected_ < 0.01; cluster’s peak locations are in Montreal Neurological Institute (MNI) coordinates, locations for the cerebellum subdivisions are from MNI-SUIT space (AFNI-SUIT atlas^92^), cluster size in number of voxels, XL(R) = X left(right), X = CM, LB, SF.

Subdivision	x	y	z	Brain region	cluster size	T-score
**CML**	−24	−6	−14	left Amygdala	71314	>13
	22	−80	28	right Superior occipital gyrus	477	−5.9
	−14	−92	24	left Superior occipital gyrus	397	−5.9
**CMR**	24	−2	−14	right Amygdala	24511	>15
	−8	90	36	left cuneus	2277	−6.6
	−16	−68	−46	left cerebellum (VIIIa)	2244	6.8
	2	58	28	left superior frontal gyrus	1488	7.7
	0	−4	48	cingulate Gyrus	1394	7
	16	−72	−52	right cerebellum (VIIb)	533	7.6
	4	−62	32	right precuneus	355	6.5
	50	−46	−24	right inferior temporal gyrus	293	5.7
**LBL**	−28	−8	−20	left amygdala	58058	>13
	0	58	36	superior frontal gyrus	1383	8.8
	50	−50	56	right inferior parietal lobule	856	−7.6
	4	34	38	right medial frontal gyrus	658	−7
	2	52	−16	right Mid. Orbital gyrus	629	8.1
	−8	−70	34	left precuneus	525	−7.6
	−38	−56	46	left inferior parietal lobule	473	−7.6
	2	−22	30	right cingulate gyrus	454	−8.7
	40	10	24	right inferior frontal gyrus	398	6.1
	58	28	2	right inferior frontal gyrus	307	6
**LBR**	30	−6	−22	right amygdala	42326	>13
	0	58	−10	medial frontal gyrus	2564	9.2
	32	−82	−36	right cerebellum (Crus I)	931	8.7
	50	−46	42	right inferior parietal lobule	870	−6.6
	−26	−82	−36	left cerebellum (Crus I)	665	9.2
	2	32	40	right medial frontal gyrus	591	−7.6
	−6	−72	38	left precuneus	387	−6.6
	0	−26	28	cingulate gyrus	310	−6.6
**SFL**	−16	−6	−18	left amygdala	68153	>13
	38	−52	−32	right cerebellum (Crus I)	3118	−11.5
	30	56	18	right superior frontal gyrus	2793	−8.8
	−32	58	16	left middle frontal gyrus	1434	−9.1
	2	28	40	right medial frontal gyrus	1286	−9.1
	54	−44	42	right inferior parietal lobule	1087	−10.2
	−40	−30	−10	left inferior temporal gyrus	793	−7
	−8	−68	38	left precuneus	733	−8.4
	−50	−52	40	left inferior parietal lobule	641	−7.3
	36	−46	0	right middle temporal gyrus	623	−7.4
	4	−24	28	right cingulate gyrus	451	−8.5
	14	−12	6	right thalamus	308	−7.5
**SFR**	20	−6	−20	right amygdala	65553	>13
	−40	−60	−30	left cerebellum (Crus I)	1579	−9.7
	2	38	22	right anterior cingulate	1541	−8.4
	−30	54	24	left middle frontal gyrus	1335	−8
	30	56	28	right superior frontal gyrus	1166	−7.7
	26	−70	−26	right cerebellum (VI)	1050	−8.8
	56	−44	36	right supramarginal gyrus	916	−8.7
	−54	−48	36	left inferior parietal lobule	895	−9.4
	2	−22	28	right cingulate gyrus	639	−10.5
	−8	−72	40	left precuneus	303	−8.5
	46	18	0	right inferior frontal gyrus	302	−6.6
	12	−70	42	right precuneus	297	−10
	−14	−12	10	left thalamus	290	−7.2

**Table 2:** Locations of the significant clusters obtained from the direct comparison of the functional connectivity pattern of each amygdala subdivision with the average connectivity pattern of the other two subdivisions. Results are corrected for multiple comparisons, *p*_corrected_ < 0.01; cluster’s peak locations are in MNI coordinates, cluster size in number of voxels, XL(R) = X left (right), X = CM, LB, SF.

Subdivision	x	y	z	Brain region	cluster size	Z-score
**CML** > **LBL** + **SFL**	−18	−8	−10	left amygdala	33154	>13
	10	−28	74	right paracentral lobule	20900	−10.0
	0	26	32	left middle cingulate cortex	4865	10.6
	−36	54	20	left middle frontal gyrus	1691	8.0
	−68	−22	24	left postcentral gyrus	1656	8.7
	30	40	28	right middle frontal gyrus	1398	6.5
	68	−32	36	right supramarginal gyrus	1195	6.8
	28	−6	−20	right hippocampus	817	−11.2
**CMR** > **LBR** + **SFR**	48	−74	2	right middle temporal gyrus	23328	−8.2
	30	−4	−24	right amygdala	6105	<−13
	−52	−48	8	left middle temporal gyrus	2288	−7.3
	60	−4	−10	right superior temporal gyrus	2129	−7.2
	−36	−46	−34	left cerebellum (VI)	2122	7.4
	2	42	20	left anterior cingulate cortex	1498	7.1
**LBL** > **CML** + **SFL**	−34	−4	−26	left amygdala	12381	>13
	0	4	30	left anterior cingulate cortex	1870	−7.0
	30	−84	20	right middle occipital gyrus	1487	6.0
	−18	−92	16	left middle occipital gyrus	1079	5.4
**LBR** > **CMR** + **SFR**	34	−2	−24	right amygdala	2731	>13
	−30	−8	−20	left hippocampus	1007	12.3
**SFL** > **CML** + **LBL**	−14	−4	−18	left amygdala	13575	>13
	−30	−70	−24	left cerebellum (VI)	9287	−10.1
	0	−20	62	left paracentral lobule	8517	10.1
	−30	54	16	left middle frontal gyrus	7197	−9.2
	0	40	−22	left rectal gyrus	2460	9.9
	56	−66	22	right middle temporal gyrus	1549	7.1
	−24	−80	34	left superior occipital gyrus	1470	6.9
	16	−104	−4	right calcarine gyrus	1390	−6.1
	0	−54	18	left precuneus	1186	7.1
	58	−44	36	right supramarginal gyrus	922	−7.6
	−62	−42	30	left supramarginal gyrus	832	−6.7
**SFR** > **CMR** + **LBR**	12	−20	76	right paracentral lobule	13240	10.1
	14	−2	−16	right amygdala	11217	>13
	−10	−54	6	left calcarine gyrus	3912	8.3
	−20	−82	−30	left cerebellum (Crus I)	1793	−8.8
	26	−70	−26	right cerebellum (VI)	1517	−8.1
	−10	30	26	left anterior cingulate cortex	1428	−6.7
	−12	−16	10	left thalamus	885	−7.1
	−56	−48	36	left inferior parietal lobule	828	−7.3
	40	24	10	right inferior frontal gyrus	803	−6.4

The LB nuclei was positively correlated with the superior, medial and inferior frontal gyri, precentral gyrus, paracentral lobule, middle temporal gyrus and cerebellum, and negatively correlated with the parietal lobule, precuneus and cingulate gyrus (Fig. 2, Table 1). Further, when compared with the circuit strength from the other two amygdala subdivision, the LB seems to be uniquely associated with inferior and middle temporal gyrus and middle occipital gyrus (Fig. 3, Table 2). Pairwise comparisons also show unique correlations of LB with the inferior temporal gyrus and the middle frontal gyrus when contrasted with CM and with cerebellum, and the middle frontal gyrus and the inferior parietal lobule when compared with SF (Fig. 1S, Table 1S).

Activity in the SF correlated with signal fluctuations in the orbitofrontal cortex, posterior insula, olfactory cortex, precentral gyrus, paracentral lobule and posterior cingulate, and was anti-correlated in the anterior cingulate cortex, superior and middle frontal gyrus, cerebellum, parietal lobule, inferior temporal gyrus and precuneus (Fig. 2, Table 1). Unique connectivity path of the SF subdivision is observed at the paracentral lobule, posterior cingulate cortex (PCC) and orbitofrontal cortex (OFC) when contrasting with the combined correlation results for the other two subdivisions (Fig. 3, Table 2).

Pairwise comparisons show that the connectivity strength of the SF subdivision is higher when compared with CM based circuits in the occipital lobe, PCC, precentral and postcentral gyrus, paracentral lobule, superior frontal gyrus and OFC and higher than the connectivity path of the LB at medial and inferior frontal gyrus, precuneus, PCC, precentral and postcentral gyrus, middle temporal gyrus, precentral gyrus and OFC (Fig. 1S, Table 1S).

### Connectivity across sessions

A two-way ANOVA (session x seed location), comparing the connectivity pattern obtained for each amygdala subunit and for each session (subseries), revealed a main effect of the seed location in the striatum, insula, OFC, ACC, superior medial gyrus, occipital gyrus, medial, middle and inferior frontal gyrus, cingulate gyrus, inferior parietal lobule, cerebellum, superior and middle temporal gyrus (Fig. 3SA, Table 3). There was no main effect of session and no interaction between seed location and session. ICC test-retest results of brain connectivity data also show fair to moderate reliability for the functional connectivity across sessions (ICC > 0.4). Intersession consistency was highest from the FC maps obtained for the SF, followed by those from CM and LB (Fig. 3SB).

**Table 3:** Significant clusters for the main effect of seed location obtained from the two-way ANOVA. Results are corrected for multiple comparisons, *p*_corrected_ < 0.01; cluster peak locations are in MNI coordinates, locations for the cerebellum subdivisions are from MNI-SUIT space (AFNI-SUIT atlas^92^), cluster size in number of voxels.

x	y	z	Brain region	cluster size	F-score
−30	−8	−24	right amygdala	10817	>20
2	30	32	left superior medial gyrus	6615	19
−50	−72	0	left middle occipital gyrus	2498	13
16	−92	22	right cuneus	2333	12
2	38	−18	right medial frontal gyrus	1449	19
28	−84	−32	right cerebellum (Crus I)	1438	15
−40	−54	−30	Left cerebellum (Crus I)	1362	20
−28	52	16	left middle frontal gyrus	1233	13
30	54	18	right middle frontal gyrus	902	14
−58	−34	40	left inferior parietal lobule	827	9.7
−52	0	−20	left middle temporal gyrus	780	12
62	−36	36	Right inferior parietal lobule	450	10
62	−4	−10	right superior temporal gyrus	371	17
40	32	−14	Right inferior frontal gyrus	278	19
−2	−28	26	Left cingulate gyrus	270	7.9

Altogether, these results indicate a stable pattern of brain connectivity for each modularity based amygdala substructure across sessions (Fig. S2) that generally reproduced those obtained with the entire time series (Fig. 2), highlighting the consistency of the connectivity over time.

### Amygdala activation and rsfMRI

**Figure 4:**
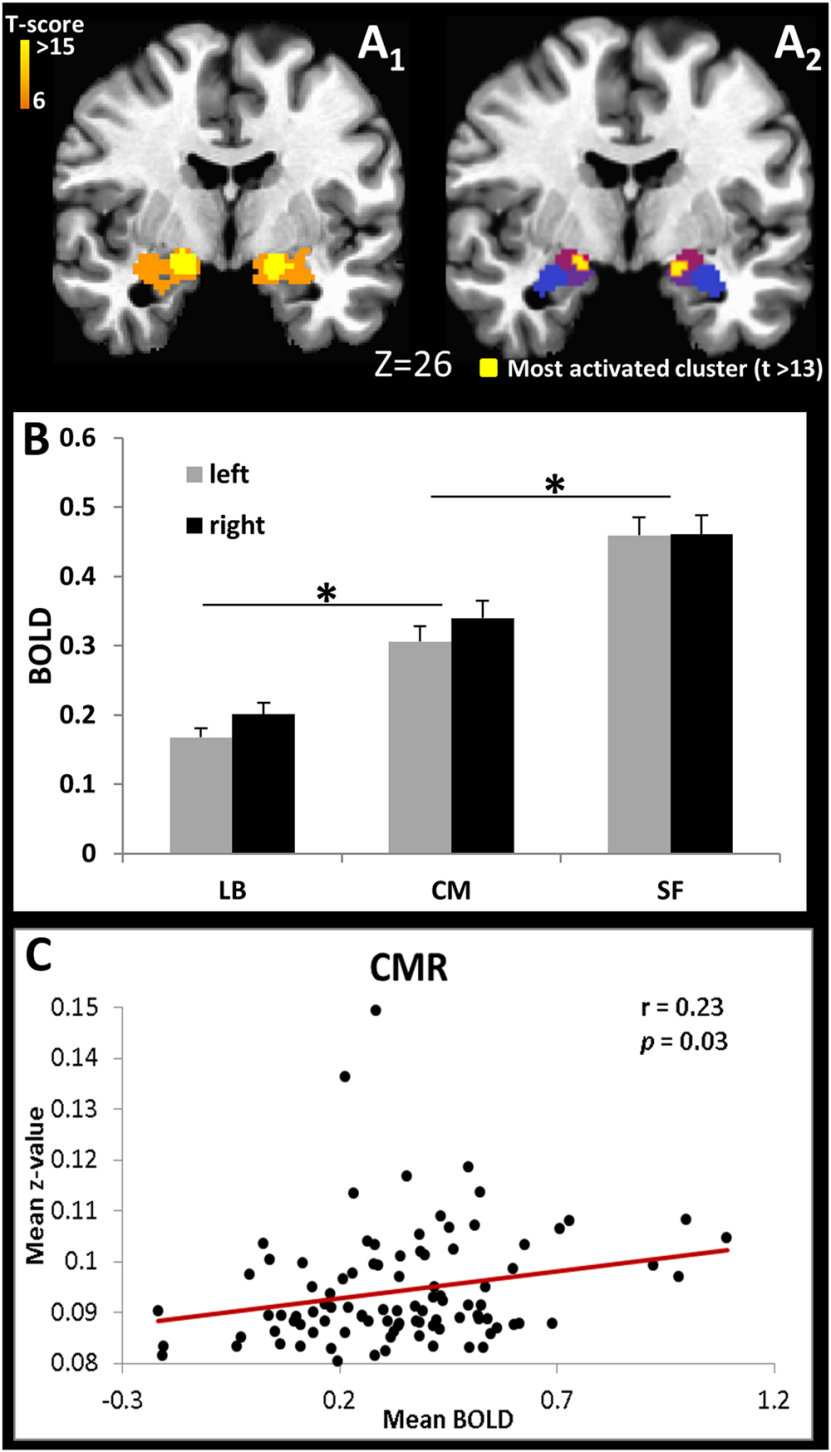
(**A**) Amygdala activation for the threshold of T ≥ 6 (**A**_1_), location of the most significant activated voxels (T > 13, peak value T = 17) (**A**_2_) overlaid over the amygdala subdivisions, CM, LB and SF (color pattern from Fig. 1), radiological convention; (**B**) Average BOLD signal for each amygdala subdivision (*p < 0.0001); (**C**) ROI analysis correlating the mean BOLD signal at the CMR with the mean positive and significant (p < 0.05) Fisher transformed correlation values of the CMR connectivity pathway.

As expected, the entire amygdala was significantly activated when subjects performed the face/shape matching task (Fig. 4A_1_). However, raising the threshold (T > 13) indicated that the most significant activated voxels were located within the CM and the SF subdivisions (Fig. 4A_2_). Average BOLD signal in each amygdala subdivision shows the LB as the least activated during the fear/anger faces task, while the superficial subdivision was the most active during the task (Fig. 4B); there were no significant differences in activation between subdivisions in the left and right hemisphere. Notably, only the CM showed significant correlation between the average BOLD signal in this amygdala subdivision and its mean connectivity strength with the entire brain **(**Figs 4C and 5S **)**, while no significant correlation between BOLD and corresponding connectivity maps was observed for the others two amygdala subdivisions.

## Discussion

By using the community structure algorithm to evaluate the topological organization of the amygdala, we identified three subdivisions, which is congruent with previous findings^16,17^, but with a method that did not require an extended meta-analysis. Moreover, modularity has shown to be very reliable, even with small datasets; the results from the HCP dataset (98 subjects) reproduced our previous pilot findings from a group of just 18 subjects, acquired in our center with different EPI sequences and different set of parameters (see Supplemental Material Fig. 4S). Additionally, our method is free of initial hypotheses related to the number of modules, which is automatically established by the algorithm based on the principle of modularity. Finally, our method was able to identify the CM and SF based only on information about node connections, despite the poor separation of these modules as illustrated in Fig. 1A, which may explain why previous data driven work^12,13^ were not able to separate the CM from SF.

Our work further supports that topological properties may have an impact on the study of structural-function relations in brain networks. Whilst it helps to understand the fundamental architecture of connections within and between brain regions, it also provides a way to elucidate how this architecture supports neurophysiological dynamics. Modularity approaches can be either top-down, such as the Newman’s method^25^, where a network is repeatedly subdivides into smaller portions until there is nothing to be gained in modularity value (Q), or bottom-up, as in Louvain’s method^26,27^, which is a hierarchical clustering approach, where nodes are progressively merged to others nodes becoming larger and larger sets of nodes, a process that is repeated iteratively until no increase of modularity is possible^28^. By using the Louvain’s method, we were able to identify amygdala modules that resemble the most accepted classification schemes for its anatomical subdivision^5^, suggesting a degree of functional segregation of these subunits that is consistent with the biological characteristics of the amygdala substructures.

Different methods have been proposed to find small communities in large networks, such as the Surprise method^29^ and Optimal Compression method^30^, each having particular strengths and weaknesses. For example the Surprise method^29^ is best suited for the study of binarized networks. However, it is vulnerable to noise errors that affect small modules, and in the case of the amygdala, located in a brain area with low SNR, it may not necessarily improve the quality of the partitions. The Optimal Compression method^30^, on the other hand, is not recommended when “*community structure are considered as statistical deviation from the null model in which the degree sequence is held constant but links are otherwise equiprobable among all nodes, wherein the modularity optimization methods by definition provides the optimal partitioning*”^30^. In contrast, by construction, the Louvain method^26^ is able to unfold a complete hierarchical community structure for the network, with each level of the hierarchy being given by the intermediate partitions found at each pass. In our work, we considered only the top level of this hierarchy, namely the final partition found by the algorithm, since no further meaningful sub-module was found in the amygdala (additional sub-modules mostly consisted of single voxels, as tested on dataset from Fig. 4S) when lower levels of modular hierarchy were evaluated.

### Regional connectivity

Temporally correlated patterns of low-frequency fluctuations during rest revealed regions of distinct functional networks associated with each of the three amygdala subdivisions, which were maintained across sessions, demonstrating the robustness and stability of our findings. Notably, their combination reproduces the previously reported^16,31^ connectivity pattern for the entire amygdala, as areas from the ventro-temporal-limbic network (described by Glerean and colleagues^31^) are identified in the connectivity pathway of the amygdala subdivisions.

Signal variation at the LB subdivision was positively correlated with activity in the superior temporal gyrus, middle frontal and precentral gyri, supporting the involvement of similar circuits in associative learning and emotion regulation^32,33^. In particular, the connection between the LB and the temporal lobes was previously observed using rsFC^6,12,16^ and probabilistic DTI^34^, and is supported by axonal tracing studies in non-human primates, where a connection between the superior temporal gyrus and the ventrolateral nucleus of the amygdala was identified^35^.

Significant correlation was found between the SF region and posterior insula, OFC, subgenual ACC, inferior parietal lobule, olfactory cortex, middle temporal gyrus and PCC. This correlation pattern suggests an involvement of SF in olfaction-related affective processes, consistent with a meta-analysis of human imaging data^17^ and rat anatomy^36,37^. Similarities of the connectivity pattern of the LB and SF mainly with the temporal lobe^38^, suggests that these two amygdaloid nuclei may share a common functional organization. Comparative architectonic studies have also suggested a separation of the SF from the CM group, assigning it to the LB group^39^, which is justified by the fact that these two nuclei also contain similar cortical –like neuronal composition^7,9^.

Despite the biological resemblance between the SF and LB, we also observed unique connectivity patterns between SF and areas of the default mode network (DMN), although anatomical projections from the amygdala to the PCC has not been verified^40^, suggesting a possible indirect connection between these regions through the sACC, as previously reported in monkeys^41^. Nevertheless, a functional connection between the amygdala and the PCC has also been reported^42^, suggesting an involvement of SF in self-awareness related emotional processes, autobiographical memory, past self-relevant stimuli and future prospection^43^. The SF was also anti-correlated with the more dorsal part of the precuneus, which is consistent with the pattern of positive correlations, previously observed, between the DMN and the ventral, but not the dorsal precuneus^44^. In addition, a decrease in regional cerebral blood flow in the PCC, medial frontal cortex and ventral but not dorsal precuneus during a working memory task^45^, suggests that dorsal precuneus (BA 7) may not be part of the DMN^46^. Furthermore, the connection between the SF and the precuneus may corroborate an amygdala-precuneus pathway that has been anatomically observed in tract tracing studies in monkeys^47,48^. Lastly, our findings showing the SF amygdala as the most activated in response to an emotional task that elicited either fear or anger, corroborates its involvement in directing attention towards affective stimuli^49^.

The significant correlation between resting activity of the CM nuclei and signal fluctuation from areas attributed to the salience network (SN), e.g., anterior insula, ACC and middle cingulate cortex (MCC)^50^, is consistent with the central nucleus involvement in facilitating attention to salient stimuli^51^. In addition, the CM was the only amygdala subdivision in which the rsFC pattern predicted activation in response to fear and angry faces, thus indicating its association with adverse feelings. Even though the SF had the highest average activation for this task, its BOLD signal did not correlate with the mean functional connectivity pattern of this subdivision. Moreover, the most activated voxels occurred not only in the SF, but also in the CM subdivision. Therefore, our findings suggest an involvement of CM and its connected areas (SN and striatum) with negative emotion and is consistent with previous findings. For example, the strongest amygdala functional connectivity within the SN is seen in those with the greatest amygdala activation to and aroused by negative pictures^52^, which is sustained by the role of the amygdala, SN and ventral striatum on the identification of the emotional significance of environmental stimuli and the production of affective states^53^. Multiple fMRI studies have also reported activation in the ACC/MCC in anger and fear emotional tasks^54^, while monkey studies show that this part of the cingulate cortex receives input from the amygdala^41,55^ and has been implicated in fear^56^. The anterior insula has also been linked with such emotional experiences as fear and anger^57^, supported by a projection from the central nucleus of the amygdala^35^. The striatum has been associated with anger^58^, such that functional connections between the CM and the striatum are enhanced during stress^59^. The CM participates not only in the expression of conditioned fear^10^ but also in the learning and consolidation of fear conditioning^60^. Experiments in rats show that lesions of the central, medial and cortical amygdala nuclei markedly increase the number of contacts a rat will make with a sedated cat, demonstrating a decrease of fear in these lesioned animals^61^. The involvement of CM in anger has also been demonstrated in cats, with stimulation of the central nucleus suppressing defensive rage, while stimulation of the medial nucleus enhances aggressive behavior^62^. Overall, our findings reinforce the previously observed participation of the CM and connected areas (SN and striatum) in fear and anger.

### Validation and reproducibility

The functional connectivity circuits for the modularity defined amygdala subdivisions reproduce the major findings obtained by Bickart *et al*.^16^ and by Roy *et al*.^6^, which used the Juelich atlas as a template. Both found that the CM nucleus correlated with the striatum and insula, that the LB complex correlated with the temporal lobe, and activity of the SF mainly correlated with activity of the OFC. Additionally, Roy and colleagues^6^ also reported laterality differences of the CM connections, as observed here, that is consistent with those previously reported^63^. For example, right amygdala lesions in rats have been shown to generate greater deficits in contextual fear than left sided lesions^64^. Previous electrophysiological studies also showed hemispheric lateralization of pain processing by CM neurons. Specifically, neurons in the left latero-capsular division of the central nucleus of the amygdala (CeLC) did not develop increased responsiveness in a rodent model of arthritis pain, while the right CeLC played a major role in the processing of prolonged nociceptive inputs and develops sensitization^65^. This suggests a tonic inhibitory mechanism from the prefrontal cortical areas over the left CeLC, which exert a top-down inhibitory influence on the amygdala^66^. In our work, the lower connectivity strength observed for the CM, besides being consistent with previous work, may also indicate a diminished inhibitory effect from the prefrontal cortical areas over the right CM, since this was the amygdala subregion most connected to these cortical areas. This may explain the unique correlation of its connectivity pattern and the activity on this area during the faces task, once more being consistent with a predominant role of the right amygdala in negative emotions. Finally, while the anatomical and functional basis for this lateralization remains unclear, taken together, these findings highlight the role of the CM on amygdala lateralization.

The functional connectivity between the LB and SF with the SMA differ from the results obtained by Roy and colleagues^6^, although they are consistent with another resting state study that identified connectivity between the LB and SF with motor areas^38^. Structural connections between the amygdala and motor areas has also been previously observed in a DTI study^34^. Studies in cats^67^ and monkeys^68^ have shown that the projections to the motor system arise from the magnocellular division of the basal nucleus of the amygdaloid complex. In addition, disruption of the amygdala-motor pathway has been suggested to be responsible for the inability of those with Autism to react to social stimuli^69^, which is consistent with data from both monkeys^70^ and humans^71^ reporting that the LB and SF subregions are especially sensitive to social stimuli. Therefore, together with previous findings, our work indicate that a direct amygdala-motor pathway might provide a mechanism by which the amygdala can influence more complex motor behaviors^34^.

Finally our results benefited from using high spatial and temporal resolution data from the HCP^22^. The high spatial resolution improves the assessment of small brain regions, such as the amygdala, by limiting partial volume effects, reducing dephasing artifacts and improving in-plane MRI signal uniformity^72^, while the high scan rate has the further advantage of minimizing the aliasing of physiological artifacts over the low frequencies of interest^73^. Even though smaller voxels always carry the disadvantage of lower SNR, it was compensated by the large sample size and multiple long rsfMRI runs.

## Conclusions

In conclusion, we have applied a community structure approach to characterize the modular organization of the amygdala using resting state fMRI data from the HCP. The anatomical and functional specificity of the results provide compelling evidence of the structure–function association between the subnuclei identified herein and histological anatomical substructures, demonstrating that graph theory based network analysis techniques can generate biologically meaningful partitions of brain structures. Finally, our findings suggest that characterization of regional modular organization may be useful to evaluate disease- or age-induced abnormalities.

## Methods

### Subjects

Analyses were performed on 100 adult healthy volunteer (46 males and 54 females; age range 22 to 36 years; mean age 29.4 ± 3.6 years), randomly selected through the 100-unrelated option from the WU-Minn Consortium HCP (WU-Minn HCP 500 Subjects + MEG2 Data Release), obtained through the Open Access agreement. Data is publicly available at the HCP online database (http://www.humanconnectome.org/documentation/S500/). All experiments were performed in accordance with the relevant guidelines and regulations of the Human Connectome Project^74^. Detailed description on the standard operating procedures of the protocol is available at the WU-Minn HCP 500 Subjects + MEG2 Data Release: Reference Manual Appendix IV. Subject recruitment procedures and informed consent forms, including consent to share de-identified data, were approved by the Washington University (WU) institutional review board^75^. The use of the HCP data was approved by the Office of Human Subjects Research Protections at the NIH. All data presented in this paper is not identifiable, only group results are presented overlaid on a MNI template.

### Data acquisition

#### MRI

Images were acquired on a 3 Tesla Skyra Siemens system using a 32-channel head coil, a customized SC72 gradient insert (100 mT/m) and a customized body transmit coil. Resting state fMRI data were acquired in four runs on two different days (two runs per day); task-evoked fMRI was acquired in two runs on the same day after completion of the rsfMRI. Both used a multi-band gradient-echo EPI sequence^22,76^ (TE/TR 33.1/720 ms, resolution 2 mm isotropic, 72 oblique-axial slices, 1200 images/rsfMRI run, 176 images/task-evoked fMRI run, MB acceleration factor = 8, BW = 2290 Hz/Pix). Within each day, acquisitions alternated between phase encoding in the right-to-left (RL) direction in one run and the left-to-right (LR) direction in the other run. Anatomical images were acquired with a high-resolution (0.7 mm isotropic) T1-weighted magnetization-prepared rapid gradient echo (3D-MPRAGE) sequence. Anatomical and functional imaging sequences covered the whole brain. Subjects were given a simple instruction to rest and keep their eyes open with relaxed fixation on a projected bright cross-hair on a dark background (presented in a darkened room)^77^.

### fMRI task – Emotion Processing

Participants performed a block design modified face-shape matching task^33^ wherein they were asked to match which of two faces/shapes presented on the bottom of the screen with the face/shape at the top of the screen; faces exhibited either an angry or fearful expression. Each of the two runs includes 6 blocks (3 face blocks and 3 shape blocks), each block was composed of 6 trials of the same task type (face or shape), with trials presented for 2000 ms and an inter-trial interval of 1000 ms. Shape blocks were presented interleaved with face blocks and each block was preceded by a 3000 ms task cue (“shape” or “face”); 8 seconds of fixation was presented at the end of each run^77^.

### Data analysis

#### Preprocessing - 1^st^ level fMRI analysis

The datasets underwent an initial preprocessing by the HCP consortium. Anatomical images were distortion corrected, co-registered and averaged across runs, AC-PC registered, brain extracted, B_1_ bias field corrected and normalized to MNI152 space. FMRI runs (rsfMRI and task-evoked) underwent gradient distortion correction, motion correction, registration to the T1w image, spatial normalization to the MNI standard space, 4D global mean-based intensity normalization, B_1_ bias field correction; rsfMRI were also subject to independent component analysis (ICA)-based artifact removal. Finally, both task and resting data were whole brain masked^23,77^. Local data processing was performed in AFNI^78^ and MATLAB (The MathWorks Inc, Natick, Massachusetts). The pre-processed task-evoked fMRI time series were smoothed (FWHM = 4 mm) to improve signal-to-noise ratio (SNR)^79^, censored for time points that exceed a Euclidean distance of 0.3 mm and then modeled in a fixed effect analysis, two runs per subject, with canonical hemodynamic responses time-locked to the shape-face epochs, contrasting face with shape condition; head motion parameters (3 translations and 3 rotations) were also entered into the model as regressors of no interest. Time-series datasets were scaled so that beta weights could be interpreted as percent signal-change. Each pre-processed rsfMRI run had the first four EPI volumes removed to ensure signal equilibrium, following by band-pass filtering (0.01–0.10 Hz) to minimize instrument induced drifts^80^ and physiological noise^81^. Multi-linear regression with the 6 time-varying realignment parameters was performed to minimize motion related fluctuations in the MRI signals. The first three principal components (using PCA) of white matter (WM) and cerebrospinal fluid (CSF) signals were also regress out to preclude non-neuronal induced signal fluctuations^82,83^. The WM and CSF masks were generated by segmenting the preprocessed high-resolution anatomical images using SPM8^84^ and down-gridding the obtained masks to the same resolution as the pre-processed functional data. Individual subject rsfMRI time series were smoothed (FWHM = 4 mm) to improve SNR^79^, and censored for time points that exceed a framewise displacement (FD) threshold >0.5 mm and the root mean square variance across voxels (DVARS) > 0.5%^85^. Time series with excessive number of time points censored (more than 30% for rsfMRI, 20% for task-evoked fMRI) were discarded; two subjects were excluded from resting BOLD and another two from the task BOLD data.

#### Modularity

Modularity analysis of the amygdala was performed using a weighted-connectivity conserving null model in fully connected, undirected networks with positive and negative weights^27^ from the Brain Connectivity Toolbox (https://sites.google.com/site/bctnet/) on the remaining 98 subjects. More specifically, Pearson correlation coefficient was computed for each voxel (node) inside of the anatomically pre-defined left and right amygdala templates in MNI space (from Jerne Volumes of Interest database^86,87^), which was resampled to 2 mm isotropic to reproduce the image resolution of the processed functional data. Individuals’ correlation matrices were then averaged across subjects and runs to obtain the final connectivity matrix, M. Following, after removing the auto-correlation values from M, a Louvain algorithm^26^, was used to find the community affiliation vector (C) corresponding to the undirected and weighted correlation matrix (M)^27^, accounting for the contribution of positive and negative edges. The algorithm finds the optimal number of clusters by aggregating the nodes in the network into groups of modules. More specifically, Louvain method starts from a set of nodes that, through subsequent passes of the algorithm, are clustered to others nodes becoming larger and larger sets of nodes (it is optimized by allowing only local changes of communities); next the found communities are aggregated in order to build a new network of communities, finally it is repeated iteratively until it reaches a number of modules that have a maximal possible number of within group connections, and a minimal possible number of between-group connections^88^, i.e., until the modularity factor, Q, is maximized^26,27^. Therefore, no initial information regarding the number of modules is needed. However, the algorithm is non-deterministic, and may produce different solutions each run, so to address this limitation, the calculation was repeated 100 times (the calculation of Q is independent in each repetition) and the maximum modularity factor, Q_M_, was registered (by considering precision of 4 decimal places only one Q value was found), then it was repeated another 100 times and another modularity factor was selected, Q_m_; these two values were compared and if they are different the latest is kept to compare with the results from the next iteration. This process was repeated until no change to the maximum Q was observed (Q_M_ = Q_m_). Note that for every step (every Q_M_ and Q_m_) the number of modules remained constant varying only their size. Next the final community affiliation, vector (C) was entered into the fine-tuning^27^ modularity algorithm, in order to refine the modules borders. Here, again the process was repeated iteratively, as before, until no change in the maximum modularity factor, Q, was observed; this allowed for the number of optimal partitions (modules) to be determined automatically. The modularity factor, Q, ranges between 0 and 1, and represents the goodness in which a network is optimally partitioned into functional modules^27^.

#### Functional Connectivity (FC) maps

Seed-voxel partial correlation was used to calculate the FC maps. The final subdivisions of left and right amygdala were used as seeds and correlated with the entire brain for each subject, regressing out the signal contribution from the neighbor seeds. The cross-correlation coefficient maps were then converted to z-score maps using the Fisher’s r-to-z transformation. Final FC maps for each subject were generated by averaging the results across runs. In order to evaluate temporal variations in the connectivity pattern generated for each amygdala seed, the original time-series were divided into four equal subseries (sessions) and FC maps were calculated for each session and averaged across runs for each subject.

One sample t-tests were performed independently on the FC maps generated for each amygdala subset. Differential assessments of the FC maps were conducted to evaluate differences in functional connectivity across amygdala subdivisions by contrasting the connectivity path of one seed against the combination of the others and by pairwise comparison (see results in the supplemental materials) using a linear mixed-effects modeling approach (3dLME)^89^. The FC pathways for the different sessions (subseries) and each amygdala subdivision were calculated with t-test, and the difference between sessions across seed location (amygdala subdivisions) was evaluated with two-way analysis of variance (ANOVA) using 3dLME^89^. Results were corrected for multiple comparisons using 3dClustSim, based on Monte Carlo simulations^90^, including voxels of the entire brain and considering a conservative imaging smoothing (FWHM) of 10 mm; statistical significance was set for *p*_corrected_ < 0.05 (see Supplemental material).

The reliability of the connectivity maps obtained for the four sessions (subseries) was also evaluated using a two-way mixed single measures intra-class correlation (ICC(3,1))^91^; specifically the between subject and residuals mean square values were computed for each voxel using the function 3dICC_REML^89^ on the FC maps generated for each amygdala subdivision, considering all sessions (see Supplemental material).

#### fMRI group analysis

One sample t-tests were performed on the beta weights generated for each subject providing a group activation map for the angry/fear faces contrasted against shapes. In addition, region-of-interest (ROI) analyses were carried out on the beta weights using the modularity results as a mask to extract the average BOLD signal in each amygdala subdivision for each subject. Results from the ROI analyses were compared with the average of the positive and significant (p < 0.05) Fisher transformed correlations obtained from the FC maps generated from seed-voxel partial correlations for each amygdala subdivision. Both analyses were carried out for the 96 subjects that survived head motion criteria for both resting and task-evoked datasets.

### Availability of materials and data

All data analyzed in this manuscript were obtained from the WU-Minn Consortium HCP (WU-Minn HCP 500 Subjects + MEG2 Data Release), which is publicly available at the HCP online database (http://www.humanconnectome.org/). Detailed description of the analysis is included in the methods section and in the supplemental material.

## Electronic supplementary material

Supplemental material
