# Supplementary material for: Graph theory reveals amygdala modules consistent with its anatomical subdivisions
Source: Sci Rep. 2017 Oct 31;7:14392. doi: 10.1038/s41598-017-14613-4 (PMC5663902; doi:10.1038/s41598-017-14613-4)
Supplement: Supplementary file 1 — Supplemental material [file 41598_2017_14613_MOESM1_ESM.pdf]

Graph theory reveals amygdala modules consistent with its anatomical subdivisions

Elisabeth C. Caparelli[1], Thomas J. Ross[1], Hong Gu[1], Xia Liang[1,2], Elliot A. Stein[1], and Yihong Yang[1]

1. Neuroimaging Research Branch, National Institute on Drug Abuse, National Institutes of Health, Baltimore, Maryland, USA
2. Research Center of Basic Space Science, Harbin Institute of Technology, Harbin, China

Corresponding author:
Elisabeth C. Caparelli, Ph.D.
Neuroimaging Research Branch
Section of MRI and Spectroscopy
NIDA-NIH
BRC, room 7A514
251 Bayview Blvd
Baltimore,MD, 21224
ph: 443-740-2288
e-mail: elisabeth.caparelli@nih.gov

Supplementary Material

Table 1S: Locations of the significant clusters obtained from the pairwise comparisons between the functional connectivity maps obtained for the amygdala subdivisions. Results are corrected for multiple comparisons, $p_{corrected} < 0.01$; cluster's peak locations are in MNI coordinates, location for cerebellum subdivision from MNI-SUIT space (AFNI-SUIT atlas (Diedrichsen et al. 2009)),cluster size in number of voxels, XL(R) = X left (right), X = CM, LB, SF

Subdivision	x	y	z	Brain region	cluster size	Z-score
**CML>LBL**	-20	-10	-12	left amygdala	38751	>13
	30	-6	-20	right amygdala	2910	-12.7
	-38	-54	46	left inferior parietal lobule	1339	8.2
	36	-24	56	right precentral gyrus	1124	-7
	14	-26	74	right precentral gyrus	771	-6.8
	-48	-22	58	left postcentral gyrus	607	-5.7
	-40	42	12	left middle frontal gyrus	601	5.7
	14	-100	-2	right cuneus	597	6.6
	-10	-66	34	left precuneus	574	6.3
**CML>SFL**	-20	-10	-10	left amygdala	54076	>13
	-46	-74	6	left middle occipital gyrus	3756	-8.4
	4	18	38	right middle cingulate cortex	3532	10.5
	0	40	-22	left orbital frontal gyrus	2217	-10.1
	26	48	20	right superior frontal gyrus	2152	8.5
	-66	-22	24	left postcentral gyrus	1397	7.1
	68	-32	38	right inferior parietal lobule	1223	7.4
	-8	-68	38	left precuneus	782	8.2
**LBL>SFL**	-32	-4	-26	left amygdala	9198	>13
	0	-20	62	left medial frontal gyrus	5048	-7.4
	-28	54	16	left middle frontal gyrus	2472	6.8
	-22	-86	-36	left cerebellum (Crus II)	1456	6.7
	26	-72	-30	right cerebellum (Crus I)	1339	8

	28	48	16	right middle frontal gyrus	1041	6.9
	-34	-84	40	left precuneus	746	-5.9
	52	-66	28	right middle temporal gyrus	588	-5.6
	54	-42	32	right supramarginal gyrus	491	6.1
	-64	-48	28	left supramarginal gyrus	427	5.7
	-38	32	-12	left inferior frontal gyrus	317	-9.3
	18	10	66	right superior frontal gyrus	308	5.6
**CMR>LBR**	42	-64	-14	right inferior occipital gyrus	9815	-6.9
	32	-4	-26	right amygdala	4786	<-13
	-30	-6	-24	left amygdala	977	-12
	-22	-64	-26	left cerebellum (VI)	424	6.7
	46	-6	-44	right inferior temporal gyrus	394	-5.7
	-28	30	48	left middle frontal gyrus	386	-5.9
	0	40	14	left anterior cingulate cortex	311	5.6
	-40	-40	58	left inferior parietal lobule	302	-4.6
	2	-24	68	right medial frontal gyrus	293	-4.5
**CMR>SFR**	24	-4	-22	right amygdala	40272	<-13
	-20	-82	-30	left cerebellum (Crus I)	3910	7.9
	2	38	18	right anterior cingulate	1716	7.8
	60	-34	34	right inferior parietal lobule	848	9
	-16	38	46	left superior frontal gyrus	843	-7.9
	-66	-36	38	left inferior parietal lobule	737	7.2
	4	-20	28	right middle cingulate cortex	478	6.7
	-38	50	22	left middle frontal gyrus	328	5.4
	36	46	36	right superior frontal gyrus	315	5.2
**LBR>SFR**	12	-20	76	right precentral gyrus	8470	-7.7
	14	-4	-18	right amygdala	4539	<-13
	4	-64	26	right cingulate gyrus	1533	-6.6
	24	-72	-26	right cerebellum (VI)	1231	7.2
	24	50	16	right superior frontal gyrus	804	6.1
	-28	52	24	left middle frontal gyrus	763	5.7
	-20	-84	-32	left cerebellum (Crus I)	747	8
	-56	-48	36	left inferior parietal lobule	731	6.9
	52	-66	28	right middle temporal gyrus	663	-5.9
	18	8	64	right middle frontal gyrus	548	6.6
	58	-34	36	right inferior parietal lobule	485	7
	-40	-70	34	left precuneus	330	-5.5
	-30	-6	-20	left amygdala	325	9.9

Figure **1S**: Pair-wise comparison of the FC maps obtained for the amygdala subdivisions, CM, LB and SF. MNI standard space; radiological convention; significance: $p < 0.05$ FWE corrected

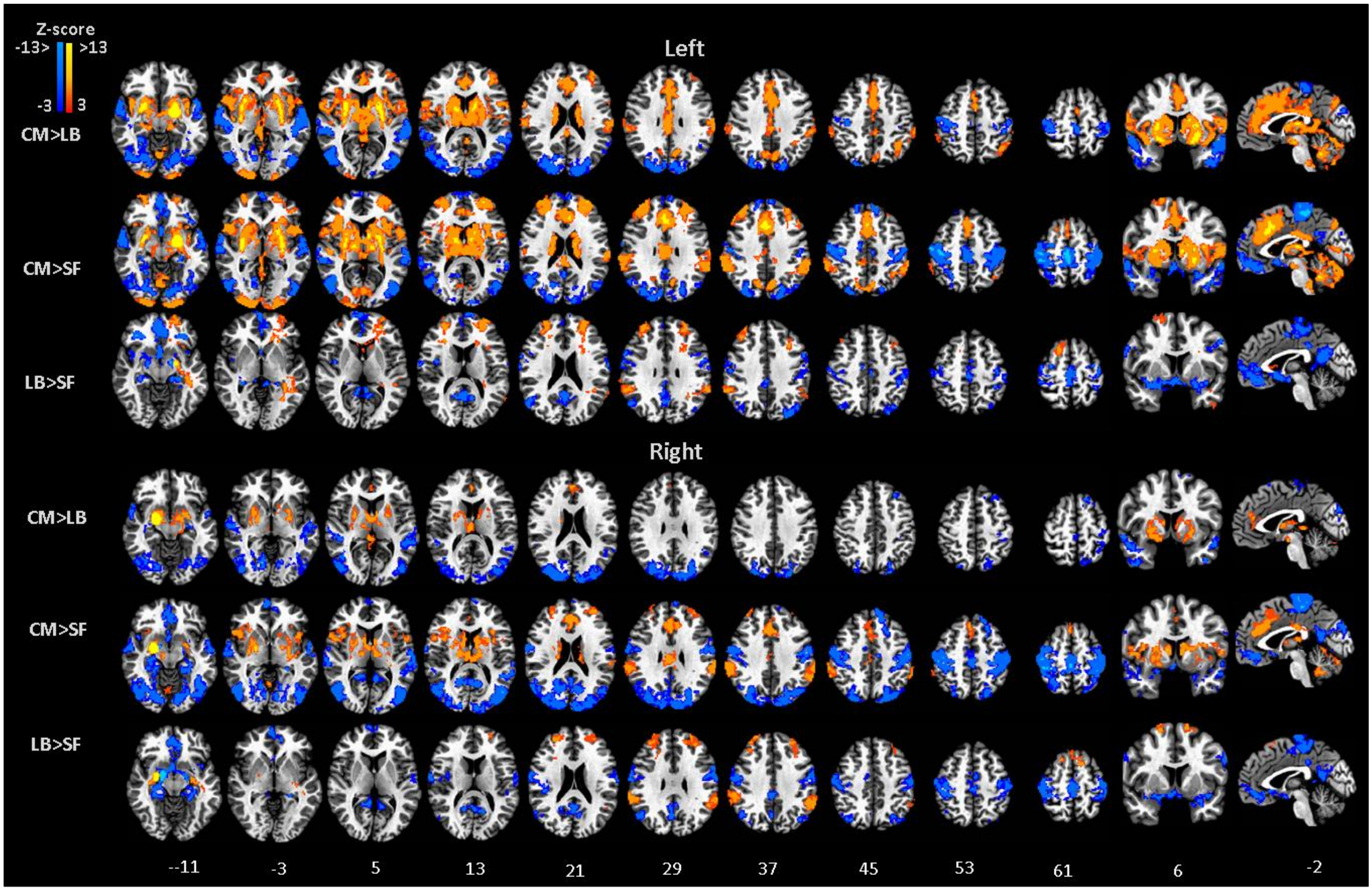

Figure **2S**: FC maps for the amygdala subdivisions, CM, LB and SF, calculated for the four different sessions. MNI standard space; radiological convention; significance: $p < 0.05$ FWE corrected

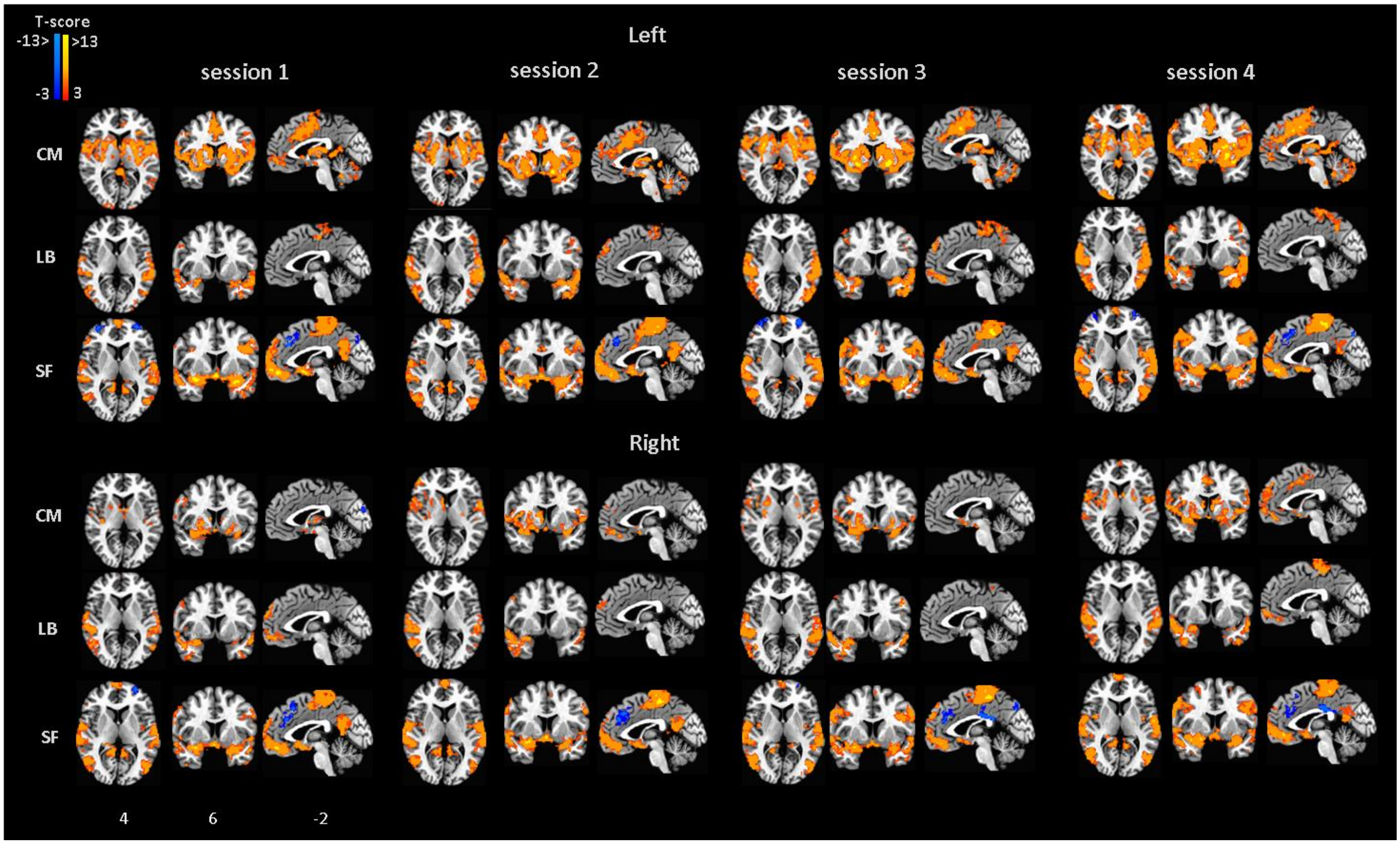

Figure **3S**: A) Two-way ANOVA results comparing the FC maps calculated for each amygdala subdivision (seed location) and for the four different sessions. A significant main effect of seed location is observed; there were no significant main effects of session or interactions. significance: $p < 0.05$ FWE corrected; B) Intraclass correlation (ICC) maps computed from the FC maps obtained for each amygdala subdivision, CM, LB and SF, calculated over the four sessions. MNI standard space; radiological convention.

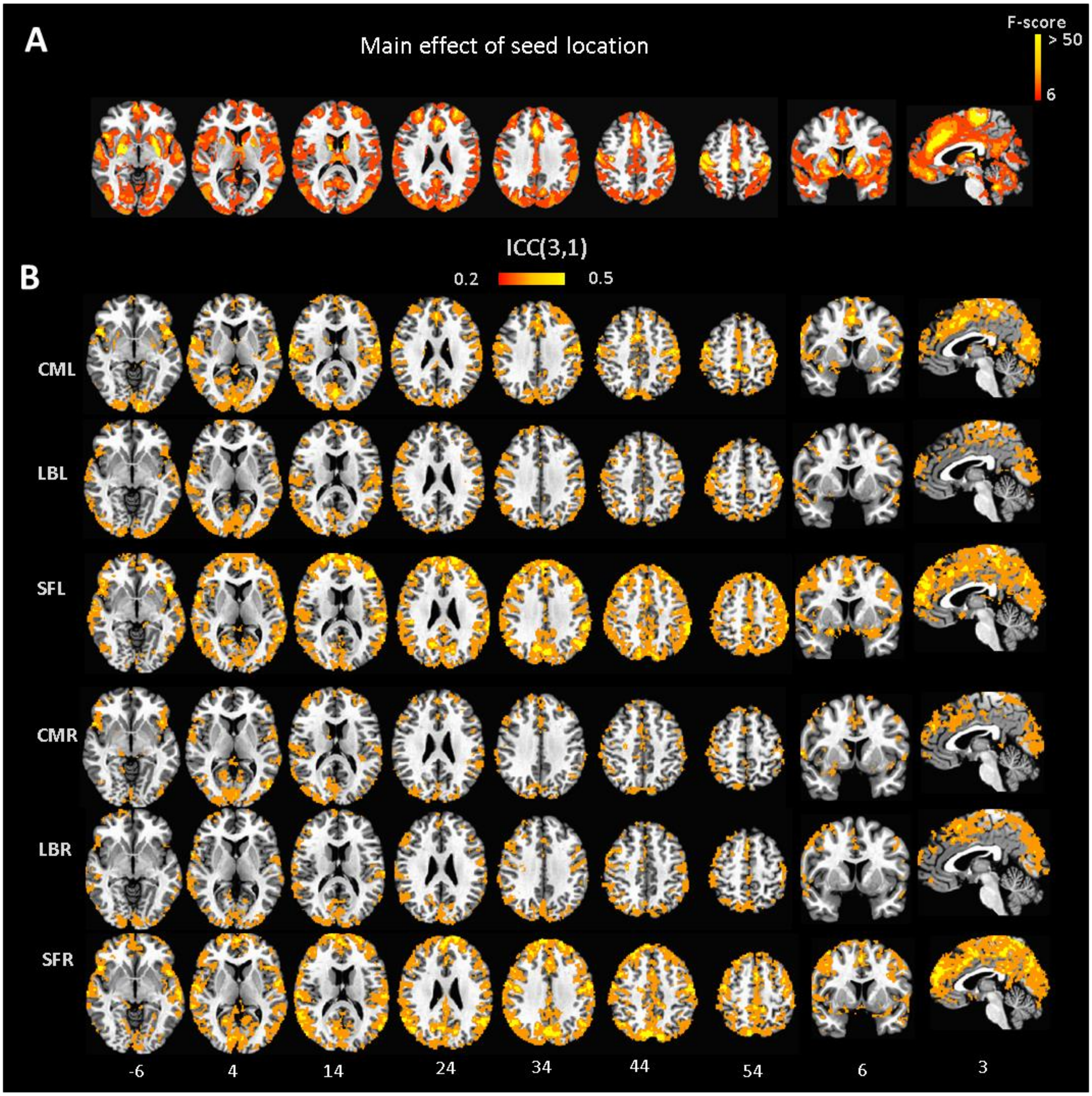

Figure **4S**: Modularity results for rsfMRI acquired with the regular EPI (Reg-EPI: TE/TR 27/2000 ms, inplane resolution 3.4×3.4×4.0 mm^3, 39 oblique slices, 240 images) and multi-band EPI (MB-EPI: TE/TR 27/500 ms, inplane resolution 3.4×3.4×4.0 mm^3, 39 oblique slices, 900 images, MB acceleration factor = 5), for 18 healthy volunteers (9 males and 9 females, 20 to 49 years of age), who gave written informed consent approved by the National Institute on Drug Abuse institutional review board panel. Images were acquired at a 3 Tesla Trio Siemens system using the 32-channels head coil.

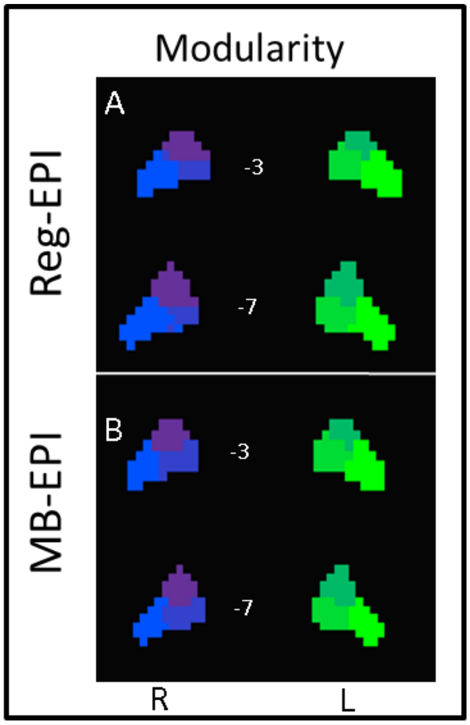

Figure 5S: ROI analysis correlating the mean BOLD signal at the CMR with the mean positive and significant ($p < 0.05$) Fisher transformed correlation values of the CMR connectivity pathway (as Fig. 4C), but removing the two possible outliers ($z>0.13$).

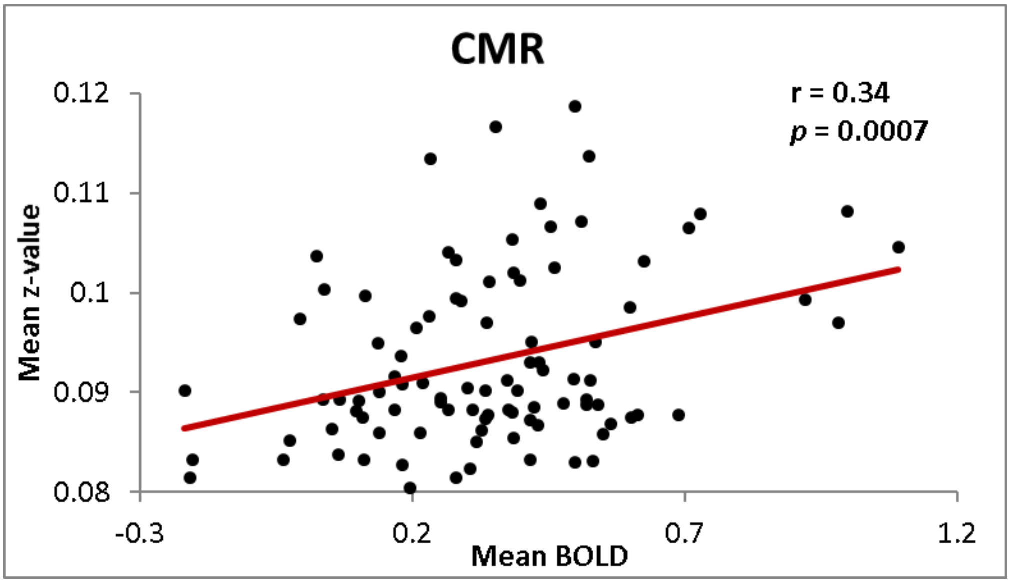

References:

Diedrichsen J, Balsters JH, Flavell J, Cussans E, Ramnani N (2009) A probabilistic MR atlas of the human cerebellum Neuroimage 46:39-46 doi:10.1016/j.neuroimage.2009.01.045
